# Dual-Role Peptide with Capping and Cleavage Site Motifs in Nanoparticle-Based One-Pot Colorimetric and Electrochemical Protease Assay

**DOI:** 10.1021/acsomega.3c00771

**Published:** 2023-06-09

**Authors:** Tamás Szabó, István Bakos, Barbara Vrbovszki, Itthipon Jeerapan, Péter Pekker, Judith Mihály, Krisztina Németh, Joseph Wang, Zsófia Keresztes

**Affiliations:** † Research Centre for Natural Sciences, Magyar tudósok körútja 2., 1117 Budapest, Hungary; ‡ Laboratory of Nano-Bioelectronics, Department of Nanoengineering, Jacobs School of Engineering, University of California San Diego, La Jolla, California 92093, United States; § Division of Physical Science and Center of Excellence for Trace Analysis and Biosensor, Prince of Songkla University, Hat Yai 90110, Thailand; ∥ Nanolab, Research Institute of Biomolecular and Chemical Engineering, University of Pannonia, Egyetem u. 10., 8200 Veszprém, Hungary

## Abstract

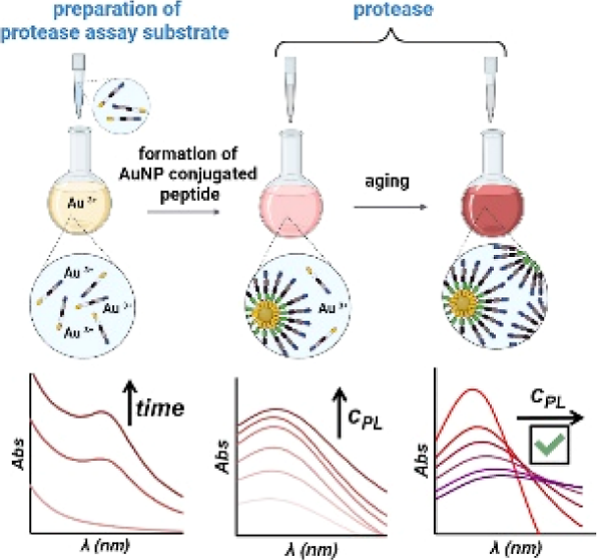

A new method for enzyme substrate assembly and its use in proteolytic enzyme assays with colorimetric and electrochemical detection is presented. The novelty of the method is the use of dual-function synthetic peptide containing both gold clustering and protease-sensitive moieties, which not only induces the simple formation of the peptide-decorated gold nanoparticle test substrates but also allows for the detection of proteolysis in the same batch. Protease-treated nanoparticles with a destabilized peptide shell became more prone to electroactivity, and thus, the model enzyme plasmin activity could be quantified with stripping square wave voltammetry analysis as well, giving an alternative method to conduct aggregation-based assays. Spectrophotometric and electrochemical calibration data proved to be linear within the 40–100 nM active enzyme concentration range, with possible extensions of the dynamic range by varying substrate concentration. The simple initial components and the ease of synthesis make the assay substrate preparation economic and easy to implement. The possibility of cross-check analytical results with two independent measurement techniques in the same batch greatly increases the applicability of the proposed system.

## 1 Introduction

Proteolytic enzymes, catalyzing peptide bond cleavage in proteins, have important regulatory roles in a wide range of normal physiological events, and in many cases, they can be considered also as biomarkers in pathological processes. Proteolysis can be quantified via commercially available homogenous assays that are mostly based on engineered peptide sequences, labeled with chromogens. Demands for the simplification of the assay protocols and facile production of substrates have resulted in the emergence of alternative signal-forming systems, such as nanomaterial-based colorimetric assays.

For their characteristic feature, the well-distinguished localized surface plasmonic resonance (LSPR),^1−3^ gold nanoparticles (GNPs) are widely used for colorimetric platforms and assays. The stabilized and the aggregated forms of GNPs in a colorimetric assay can be distinguished by the wavelength of the absorbance maximum (λ_max_) of the LSPR. In the aggregated state, λ_max_ is located at longer wavelengths due to the coupling of LSPRs of individual GNPs being close to each other. In most colorimetric assays, GNPs go from separate to aggregated state during assaying (Table S1, column “D”); in other words, the color of the sample shifts from red to blue,^4−8^ which, in the case of protease assays, correlates with the activity of the enzyme.

The stability of GNPs is generally assured by a surface-bound, electrically charged organic layer, which is prone to alterations due to enzyme activity (Table S1, column “F”). Natural proteins and synthetic, short oligopeptides are used as substrates of proteases; however, the latter is preferred due to their precisely defined structure (Table S1, column “B”). The major objectives of the design of synthetic substrates have been established^9−12^ and are as follows: (1) peptides must bind to GNPs (with −SH and/or −NH_2_ functionality), (2) they have to stabilize the nanoparticles electrostatically and/or sterically, and (3) peptides have to be susceptible to cleavage by the protease at the target recognition site.

Aggregation of GNPs can be induced by crosslinking or neutralization of the charged peptide layers via direct enzymatic cleavage or the adsorption of cleaved fragments (Table S1, column “F”).

The first GNP-based colorimetric protease enzyme activity assay was developed by Guarise et al.;^13^ since then numerous other protease assays have been published, e.g., thrombin, anthrax lethal factor, matrix metalloproteinase-1 (MMP-1, interstitial collagenase), MMP-2 (72 kDa type IV collagenase or gelatinase-A), MMP-7 (matrilysin), MMP-9 (92 kDa type IV collagenase or gelatinase-B); thermolysin—a bacterial MMP, trypsin, caspase-3; Botulinum toxin type A (a bacterial endopeptidase), DPP-IV dipeptidyl peptidase (serine exopeptidase), β-secretase, immunoglobulin A1 protease (IgA1P, a bacterial protease) (Table S1, column “A”).

Strategies regarding the preparation of the GNPs and the realization of the transition between the free and aggregated states of GNPs are numerous (Table S1). Basic GNPs are usually synthesized via methods related to Turkevich^14^ or Frens^15^ (Table S1, column “C”). Their functionalization involves the conjugation of enzyme substrates on their surfaces through ligand exchange and several centrifugation and cleaning steps. The assaying methodology, i.e., the number and order of the steps of the assay developing can be different (Table S1, column “E”). One-step detection requires only the addition of the enzyme to the previously functionalized GNPs, and thus, the activity of the protease is directly measured by its effect on the stabilizing layer of the GNPs. The two-step or mix-and-detect assays are not triggered by the enzyme but by the product of the enzymatic cleavage. This strategy requires at least two physically separated steps, i.e., the enzymatic cleavage process, and the addition of the cleavage product to GNPs, which results in aggregation and corresponding color change. Since both one- and two-step methodologies involve numerous preparation/assaying steps (Table S1, column “G”), a more convenient procedure is desirable.

The aggregation-related characteristics of metallic NPs can also be investigated with electrochemical methods such as anodic stripping voltammetry (ASV). In the case of regular stripping methods, the metallic content is first deposited onto the electrode from its dissolved form via an accumulation step, and then, the metal is dissolved during an anodic treatment.^16^ Alternatively, in the ASV investigation of NPs, the NPs themselves make contact with the electrode, via immobilization or collision, and this is followed by their anodic dissolution. From the resulting stripping voltammogram, information can be extracted regarding the quality of the protective shell,^17−21^ the size,^17,22−28^ and aggregation rate^19,27,29−31^ of the NPs. This analytical technique has been reviewed lately by Pattadar et al.^32^ This versatile method, however, has not been used in aggregation-based colorimetric protease assays yet.

**Scheme 1:**
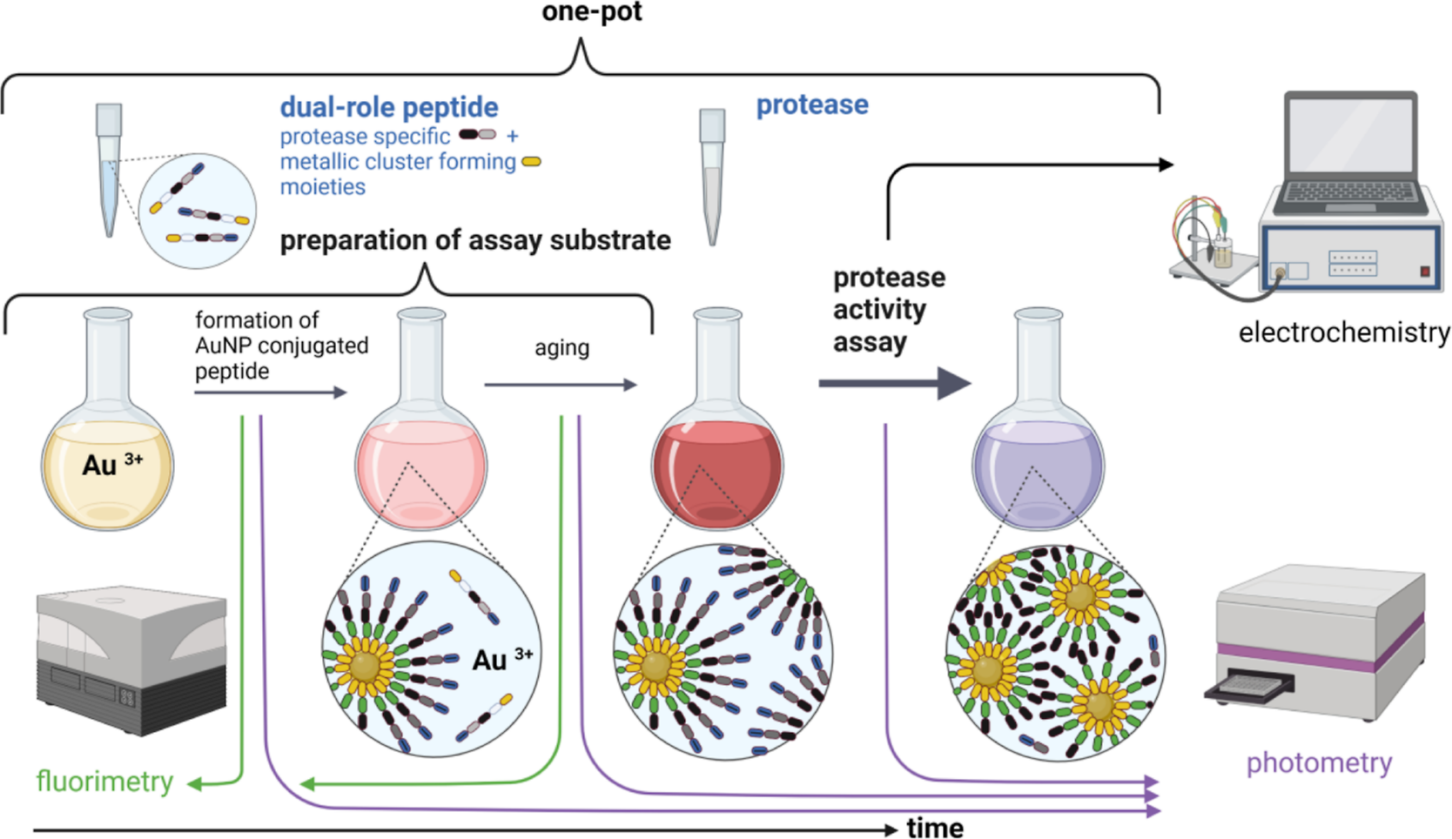
Illustration of the One-step Preparation Procedure of pGNPs, Their Aggregation Mechanism, and the Indication of Investigation Methods of pGNPs’ Aging and Aggregation

In this work, we show how a colorimetric sensing assay can be simplified to a reduced number of preparatory and assaying steps (Scheme 1), compared with the already presented examples in the literature (Table S1). The preparation has only one step, without any purification: a one-pot, peptide-assisted GNP synthesis by using a synthetic peptide with combined cluster forming-, stabilizing-, and protease substrate nature. Peptides with more than one functionality have already been introduced to GNP preparation, where the peptides provided the assay GNPs with recognition affinity toward antibodies.^33^ In our development, the protease-sensitive peptide sequence supports an aggregation-inducing enzyme reaction, which is realized in a one-step assay. In this study, plasmin was used as a model enzyme, a serine protease present in blood, with the essential role in thrombolytic dissolution of fibrin clots.^34−37^ Plasmin, as an endogenous enzyme, also infiltrates naturally into milk from blood, affecting milk quality and causing either beneficial or detrimental effects on dairy products.^38^

To the synthetic peptide structure, we introduce the pairing of a capping-^39^ and a protease-sensitive sequence. In our experiments, we have used the ”(NH_2_)CCYGGTFKGGGGGGR(COOH)” sequence, which contained the Cys-Cys-Tyr end as the complex forming and reducing unit and the Thr-Phe-Lys as the specific plasmin-cleavage unit.

Time dependence of nanoparticle formation and the effect of aging of the sample batch have been proved to be important, concerning the functionality of the peptide-stabilized GNPs (pGNPs). As a complementary and new method to colorimetric assays, we leverage ASV of pGNPs for the quantification of enzyme-induced aggregates. As will be illustrated in the following sections, the single-step mixing of a dual-function peptide and gold salt results in the simple production of pGNPs, which are convenient substrates for colorimetric and electrochemical protease assays.

## 2 Experimental Section

### 2.1 General

In all cases, 18.2 MΩ resistance MilliQ water was used. All glassware was thoroughly cleansed before use with 50 mM KOH and 25% H_2_O_2_ (30%, AnalR Normapur, VWR Chemicals, France) solution. 10 mM phosphate buffered saline (PBS) solution of pH = 7.4 was used for dilution of the pGNP sample and dialysis of plasmin.

### 2.2 Preparation of pGNPs

Materials: gold(III)chloride trihydrate (p.a. ≥ 99.5%; Carl Roth + Co. KG; Karlsruhe, Germany); synthetic peptide “(NH_2_)CCYGGTFKGGGGGGR(COOH)” (purity: 95.072%; Lot# GTA32516-1-1210; NovoPro Bioscience Inc., Shanghai, China); 1 N sodium hydroxide aqueous solution (AVS itrinorm, VWR Chemicals, France).

With the preparation of pGNPs, we used a formerly published method by Xie et al., with alterations.^40^

For the preparation of pGNP1 samples, equal “*V*_x_” volumes of 5 mM (0.2% w/w) aqueous Au-salt solution and 0.538 mM (0.074% w/w) aqueous peptide solution were mixed and shaken with a vortex mixer (Vortex Mixer, VELP Scientifica, Europe). Right after, the sample was shaken and incubated in a pre-heated incubator (TH15/KS15 Edmund Bühler GmbH, operated at 100 rpm) at 37 °C for 20 min. Subsequently, to adjust a slightly alkalic (∼8.5–9) final pH to the pGNPs, 1.0 M NaOH solution was added in a volume of 0.05 *V*_x_, and the mixture was vortexed again, kept incubated, and shaken for 1776 h (74 days). A pGNP10 sample with 10-fold peptide concentration was also prepared. Before use, the samples were left to cool down to room temperature (RT) and kept still and intact. Samples for characterization, aging monitoring, and enzyme testing were taken during and after incubation. For the optimization of the enzyme treatment, when it was necessary, pGNP samples were diluted with PBS solution.

### 2.3 Enzyme Treatment of pGNPs

Pre-activated, stabilized plasmin was used for the experiments (5U stock, from bovine plasma; containing 3.2 M ammonium sulfate solution as a stabilizer; REF 10602370001; LOT# 36608220; Roche Diagnostics GmbH, Mannheim, Germany). Dialysis of the enzyme was carried out by 10–250 μL capacity dialization vessels (Pur-A-Lyzer Mini 6000 Dialysis Kit, Sigma-Aldrich) against PBS solution.

Previously, the actual activity of plasmin was measured to be 30 μM. From the purified stock solution, dilution series were prepared with PBS and then were added to pGNP samples in the optical plate wells to obtain 0–500 nM solutions, with regard to plasmin. The samples were mixed and kept at RT before analysis.

Inactive plasmin (plasmin was kept in boiling water bath for 2 h), bovine serum albumin (laboratory reagent grade, fraction V, Fisher Scientific, UK), and β-lactoglobulin (≥90% PAGE, Sigma-Aldrich, USA) were used as reference assaying materials.

### 2.4 UV–Visible Spectrometry and Spectrofluorimetry

All absorbance and fluorescence measurements were carried out with a Varioskan Flash plate reader (Thermo Scientific, data collection: SkanIt, default software of the instrument), with translucent and black 96-well polystyrene plates. Samples were shaken/stirred before portioning into the wells. Photometer settings included 1 nm resolution and 500 ms sampling time. In fluorescence mode, an upper exposure position was applied, and the excitation wavelength was 250 nm.

### 2.5 Transmission Electron-Microscopy

Samples for transmission electron microscopy (TEM) were prepared by drop-casting on copper TEM grids covered by a lacey carbon amorphous support film (Ted Pella, Inc.; Type-A; 300 mesh, Cu). High magnification TEM analyses were performed using a Talos F200X G2 instrument (Thermo Fisher), operated at 200 keV accelerating voltage, working in scanning TEM (STEM) mode, with a high-angle annular dark field (HAADF) detector. Lower magnification transmission electron-micrographs were taken with a Fei Morgagni 268D at 100 keV.

### 2.6 Dynamic Light Scattering and Zeta Potential Measurements

Both dynamic light scattering (DLS) and zeta potential measurements were performed with a Zetasizer Nano, Nano ZS (Malvern Instruments Ltd. Worcestershire, United Kingdom; data collection: default Malvern software) equipment with standard (Sarstedt UV-transparent disposable) and DTS1070 disposable cuvettes, respectively. The dispersion medium was water; measurements were run at 25 °C. The refractive index was 0.2 for Au nanoparticles; absorption coefficient *k* was 3.320; all other parameters (such as measurement time, position, etc.) were automatically set. Zeta measurements were carried out via the diffusion barrier method, the cells were filled with background solution, and 50 μL of the sample material was injected to the bottom of the capillary; the Smoluchowski model was set for data fitting with an F(κa) value of 1.5; other parameters were set automatically.

### 2.7 Capillary Electrophoresis

Background electrolyte (BGE) components phosphoric acid and triethylamine were purchased from Sigma (St. Louis, MO, USA) and from Merck GmbH (Darmstadt, Germany), respectively. Capillary electrophoresis was performed with an Agilent Capillary Electrophoresis 3D^CE^ system (Agilent Technologies, Waldbronn, Germany) applying DB-WAX-coated silica capillary having a 33.5 cm total and 25 cm effective length with 50 μm inner diameter (Agilent Technologies, Santa Clara, CA, USA). On-line absorption at 200 nm was monitored by a DAD UV–vis detector. The capillary was thermostated at 25 °C. Before measurements, the capillary was rinsed subsequently with distilled water for 15 min and between measurements with BGE (100 mM trimethylamine-phosphate buffer, pH 2.5) for 3 min. Samples were injected by 5 × 10^3^ Pa pressure for 6 s. Runs were performed in the positive-polarity mode with 20 kV. The plasmin cleavage assay mixture contained 100 μM peptide and 1 μM plasmin and the enzyme reactions were kept at RT (25 °C).

### 2.8 Fourier-Transform Infrared Spectroscopy

Changes in the peptide structure were followed by fourier-transform infrared spectroscopy (FT-IR). Measurements were performed by means of a Varian 2000 (Scimitar Series) FT-IR spectrometer (Varian Inc., US) using a MCT (mercury-cadmium-telluride) detector and fitted with a “Golden Gate” single reflection diamond ATR accessory (Specac Ltd., UK). The applied attenuated total reflection (ATR) technique allows for the study of aqueous solutions. 5 μL of the sample was pipetted on the top of the diamond ATR element, and the spectrum was collected immediately with 128 scans at a spectral resolution of 2 cm^–1^. All spectral manipulations (water spectrum subtraction, second derivative, etc.) were performed using the GRAMS/AI software package.

### 2.9 Cyclic Voltammetry and Anodic Square-wave Voltammetry

2D printed graphite electrodes were manufactured at Nano-Bioelectronics Laboratory, UCSD, with a high-precision semi-automatic screen printer (Model TF 100, MPM-SpeedLine Inc). The three-electrode system was printed on a polyester foil. This sensing system consisted of graphite (Ercon Inc, 7 Kendrick Rd #3, Wareham, MA 02571, USA) counter and working electrodes, whereas Ag/AgCl (Ercon Inc) was used for fabricating a reference electrode and electronic connections. Measurements were performed with an Autolab PGSTAT128N potentiostat (Eco Chemie, Netherlands), and data were collected with Nova software (ver.: 1.11).

100–100 μL of pGNP samples was dropped on the electrode system and was covered to avoid evaporation. Then, the samples were subjected to an anodic stripping square wave voltammetric electrochemical program (Figure S1) that consisted of cathodic deposition and subsequential anodic square wave voltammetry (ASWV) to reveal their electroactive gold content.

The electrochemical program starts with an applied cathodic potential −0.5 V for 120 s and continues with the SWV analysis. Electroanalytical parameters for SWV were as follows: start potential +0.550 V, end potential: +0.900 V; step potential 0.001 V; amplitude 0.025 V; frequency: 5 Hz; and scan rate 0.005 V s^–1^. This program was used for assessing the extent of aggregation of metallic nanoparticles after enzyme treatment.

The effect of enzyme treatment was also investigated with cyclic voltammetry (CV). Setups for CV were as follows: start potential +0.210 V; upper vertex potential +1.100 V; lower vertex potential −0.500 V; step potential 0.010 V; and scan rate 0.100 V s^–1^.

All applied potentials are compared to an Ag/AgCl reference electrode.

## 3 Results and Discussion

### 3.1 Preparation of Different pGNPs and Time Dependence of Nanoparticle Formation

**Figure 1:**
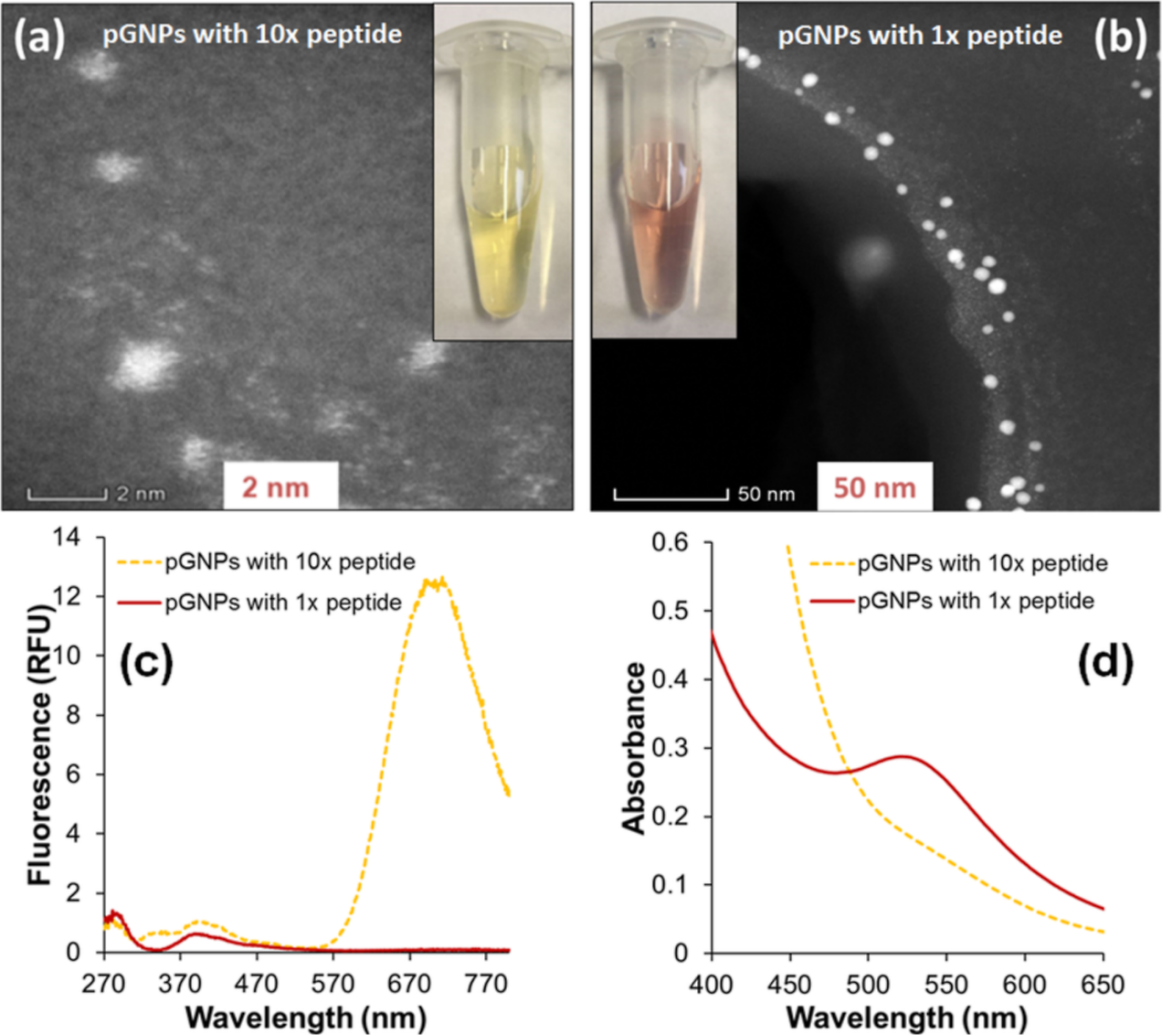
Effect of stoichiometry on the size and the spectral characteristics of pGNPs. High magnification STEM micrograph of (a) pGNP10 and (b) pGNP1 nanoparticles (spots with bright contrast), 3 days after their preparation. Inset photographs of yellow and red colored products show the macroscopic appearance of the nanoclusters and nanoparticles, respectively. (c) Fluorescence and (d) absorbance spectra of 3 days old pGNP1 (solid line) and pGNP10 (dashed line). In the case of fluorescence, λ_ex_ was 250 nm.

Different preparation stoichiometries resulted in different size GNPs. The reaction with 10-fold peptide gave pGNP10 gold clusters (subnano particles) with a small size less than 1.0 ± 0.5 nm (Figure 1a), in accordance with the mechanisms reported in previous studies.^39,40^ Meanwhile, pGNP1 samples yielded GNPs with the relatively larger dimension of 10 ± 3 nm (Figure 1b). Size data were derived from TEM images. We used UV–vis absorbance and fluorescence spectroscopy (Figure 1c,d) to reveal the photometric character of these two nanoparticle samples. We found that pGNP1 shows the presence of the plasmonic peak of the GNPs at 527 nm (Figure 1d), while its fluorescence spectrum reports only the autofluorescence of the stabilizing peptides (280 nm) and their oxidized forms (410 nm) (Figure 1c). However, pGNP10 was fluorescent with a broad emission band at 700 nm (Figure 1c), indicating fluorescent gold clusters, and lacked the LSPR peak (Figure 1d), illustrating the significant effects of different stoichiometries. As part of the development of the colorimetric and electrochemical assay, we worked with the pGNP1 (further referred to as “pGNP”) samples.

**Figure 2:**
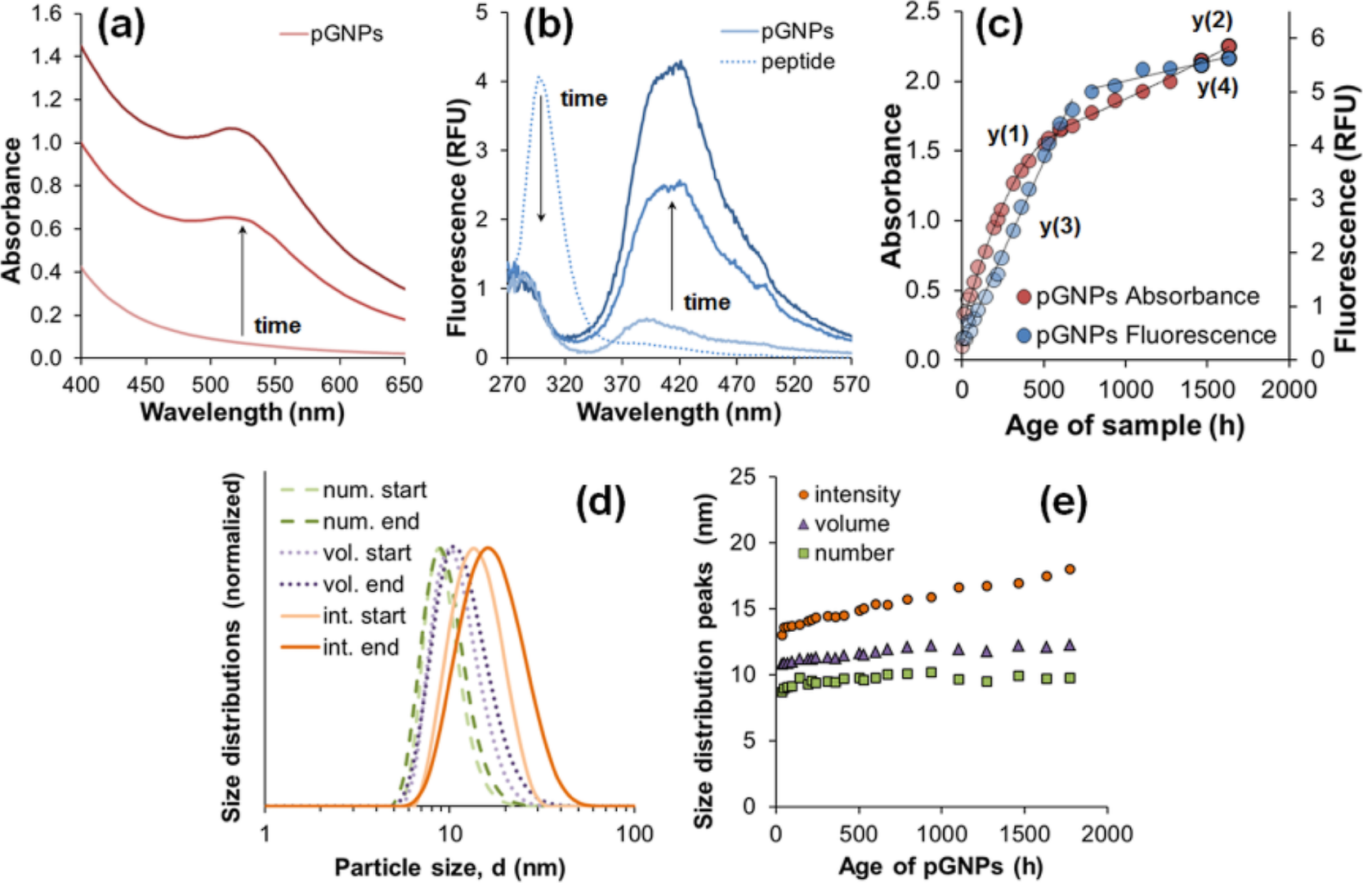
Generation and aging of pGNPs. (a) Representative absorbance spectra with evolving LSPR band with λ_absorbance max_ = 527 nm. (b) Representative fluorescence spectra of the diminishing fluorescence of peptide (dotted line) and the increased fluorescence of di-tyrosine, peaking at λ_emission max_ = 410 nm (solid lines). (c) Timely comparison of measured maxima averages of respective absorbance (red circles) data, fluorescence spectra (blue circles), and their fitted trendlines regarding a 0–1776 h long monitoring. (d) DLS-derived plots compare the starting (672 h, pale lines) and final (1776 h, dark lines) size distributions by number (dashed lines), volume (dotted lines), and intensity (solid lines). (e) Mean values of size distributions by intensity (circles), volume (triangles), and number (squares) versus the age (0–1776 h) of pGNPs.

Absorbance measurement was used to monitor the aging of the samples. In a 0–1776 h time course, an increase in the intensity of the plasmonic peak of pGNP population could be observed related to the formation of new nanoparticles (Figure 2a). Besides that, we also considered fluorescence spectra to find aging markers. The native peptide has an intensive autofluorescence at 300 nm that disappears almost instantly after the gold salt solution is added (Figure 2b). The phenomenon was discussed with another aromatic, reducing amino acid, tryptophan, by Coronato Courrol and de Matos^41^ The diminishing emission of fluorescent amino acids can be associated with static quenching or non-radiation interaction with the freshly formed reduced gold.^42,43^ The appearance of a broad, more complex band between 400 and 420 nm (Figure 2b), produced during pGNP generation, can be attributed to the fluorescence of dityrosine (di-Tyr, oxidized tyrosine), based on previous studies of protein (BSA, lyzozyme, and ovalbumin)-gold nanoclusters.^44,45^ The key role of tyrosine^33,39,46−51^ and dityrosine^39,46−51^ in gold nanocluster and GNP production via reduction of gold(III) was demonstrated by other scholars as well. The emitting moiety was also present in the native peptide (Figure 2b), but in a much lesser degree than in the GNP-containing samples.

The aging was quasi-spontaneous, and 37 °C incubation temperature was applied in order to avoid the possible negative structural impact on the peptides from higher temperatures but to slightly accelerate the reduction of GNPs. The 527 nm plasmonic absorbance band in Figure 2c shows a monotone increasing trend, seemingly producing two different kinetics. Based on the fitting equations [*y*(1) and *y*(2), Table S2], the first part of the aging lasts up to around 672 h (or 28 days), and from there, it has a more linear character. With age, the fluorescence of di-Tyr recorded at 410 nm, for the ease of handling, increases as well (Figure 2c), and its trend of 0–672 h period [*y*(3), Table S2] is similar to that of absorbance. However, the 664–1776 h interval has a moderate slope [*y*(4), Table S2]. This correlation proves that tyrosine takes part in the reduction of GNPs. The presence of physical agglomerates of pGNPs may also contribute to a slight increase in absorbance in the later stage. However, the effect of agglomeration is small, as shown in Figure 2d,e.

DLS measurements correspond to the hydrodynamic size of the particles. Figure 2d shows the starting and final size distributions of the particles in the 672–1776 h interval (Table S3). Figure 2e presents the change of size distribution maxima values during the whole period (0–1776 h). Until the age of 672 h, all three representations show some growth, standing for the generation and growth of the particles. After that, the numeric and volumetric representations reveal no or very little size change, while the intensity-related size plot reveals a growth, which is also shown in Figure 2d. Figure 2d shows a widening of all three distributions toward the larger region as well. This apparent growth could not be captured by TEM, though it can be explained by relatively few agglomerates in the sample, that also contribute to light scattering in intensity measurements. However, they do not constitute a significant multitude or volume in the sample. Polydispersity (PDI) values for pGNP samples, referring to the age of 672 and 1776 h, are 0.253 and 0.263, respectively, which confirm the stability of the product.

### 3.2 Plasmin Treatment of Peptides and pGNPs–UV–Vis Photometry

**Figure 3:**
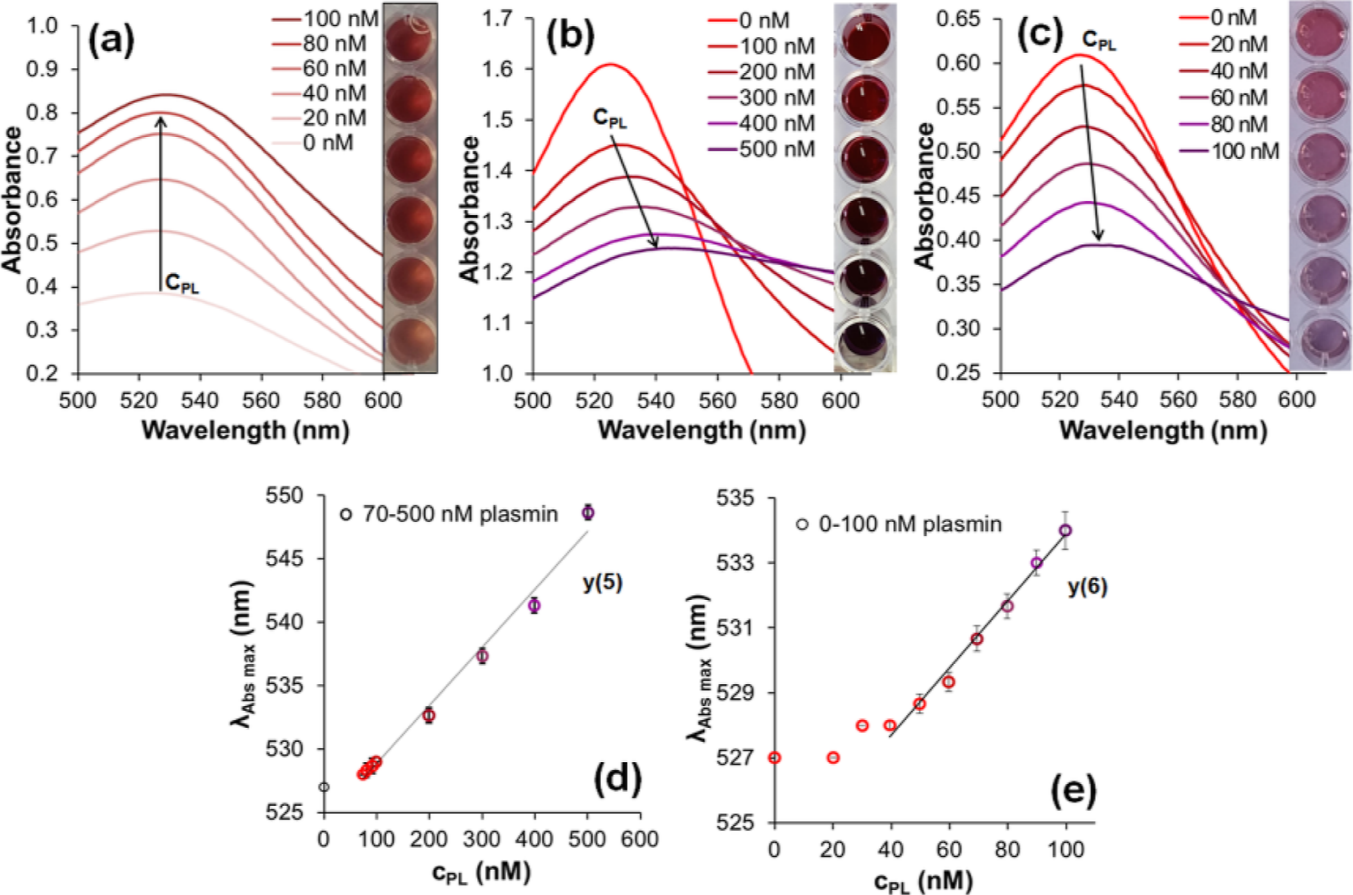
Effect of plasmin treatment on colorimetric sensing. (a) Absorbance spectra of young (5 days old), 0–100 nM plasmin-treated pGNPs after 1 day incubation. (b) Absorbance spectra of 28 days (672 h) old, 0–500 nM plasmin-treated pGNPs with no incubation. (c) Absorbance spectra of 28 days old, 2× PBS-diluted, 0–100 nM plasmin-treated pGNPs with no incubation. Inset photographs in (a–c) are illustrations of the color change of pGNPs in plate wells, following plasmin treatments. (d,e) Plots of λ_absorbance max_ values vs 0–500 and 0–100 nM plasmin concentrations, respectively. The error bars were ±1 standard deviation from triplicate measurements.

The cleavage of the substrate peptide by plasmin was proven by capillary electrophoresis (Figure S2). In terms of colorimetry, it is expected that plasmin as a target analyte destabilizes the pGNPs, causing their aggregation, which is detectable via the color change. As an approach for the quantification of GNP aggregation, the color of the pGNPs, in other words, the position of the plasmonic absorbance peak maximum was considered as the analytical signal, since the ratio *A*_max aggr_/*A*_max0_, (where *A*_max aggr_ is the absorbance maximum of the aggregated GNPs and *A*_max0_ is the absorbance maximum of the non-aggregated GNPs) was not applicable, due to the lack of two separate characteristic absorbance peaks of the aggregates and the non-aggregates, respectively (Figure S3). In spite of the expectations, in the case of our samples, particularly in the earlier period of aging, we found that the addition of plasmin rather leads to an increase of absorbance intensity than color shift, showing the effect of the sample age on the enzyme detection mechanism. Figure 3a shows the result of plasmin addition to the 5 days old, early phase pGNP sample. The plasmin addition had no instant effect, while in 1 day’s course, up to 100 nM plasmin concentration, there was a monotonic increase of absorbance, with no detectable color shift. This suggests that the pGNP formation was not fully accomplished after the 5 days reaction. Thus, the enzyme helped to reduce available gold(III) to form GNPs. Reduction of gold(III) to gold nanoclusters had been achieved through another serine protease, trypsin, as a reducing/capping agent.^52^ Plasmin indeed contains a great variety of other possible reducing moieties and was reportedly used to generate GNPs.^53^ The investigation of the interaction of trypsin and GNPs showed that GNPs may stabilize the structure of the enzyme;^54^ however, over a certain gold/trypsin ratio, the interaction leads to damage to enzyme structure and functionality.^54,55^ The proteolytic functionality of plasmin, during its assistance in GNP generation, is being compromised, and thus, at the early aging stages of pGNP samples, the assay falls short of the detection of plasmin activity.

However, the addition of plasmin results in excessive pGNP aggregation if the 672 h aging time has passed (Figure 2c; previously discussed). This clear aggregation causes a visible and measurable color shift; the change of λ_max_ can be seen, as shown in Figure 3b–e. The reliability of the older sample was proven, using a ripen sample of 28 days (or 672 h) old (Figure 3b), where further pGNP generation was negligible, but the color change was observable with relatively high (100–500 nM) plasmin concentrations. The range of plasmin sensing with the pGNP sample was linear until 70 nM (Figure 3d), but not below that. Focusing on present day’s expectations toward lower detection limits, we found that by diluting the pGNP sample twice with PBS solution, the linear sensing could be adjusted for the 0–100 nM region (Figure 3c,e), where the linearity goes as low as 30–40 nM, which is comparable to the systems published in literature (Table S1, column “H” and “I”). Diluting the original pGNP, the preparation batch could provide another linear detection range. Fitted linear regression curves (Table S4) show that the diluted pGNP sample [*y*(6), Figure 3e] is more sensitive than the undiluted one [*y*(5), Figure 3d].

As a reference for plasmin quantification, a comparison was also performed to characterize the responsiveness of pGNPs upon the addition of other biomolecules, such as deactivated plasmin, bovine serum albumin, and β-lactoglobulin (Figure S4). The representative results show that the investigated molecules do not give false positive results and that the plasmin is indeed detected by its enzymatic activity.

Once aggregation is considered as an analytical signal, light scattering, as a less frequently reported phenomenon, should also be discussed. Besides, aggregates reduce the absorbance of a GNP sample over a given measure of aggregation, and the light scattering can be so intense that the spectrum becomes uneven, wavy with various small local maxima,^4^ making it difficult to find an obvious, clear one (Figure S5). This emphasizes the importance of precision in optimizing sensing medium conditions such as age, concentration, stability, and enzyme detection parameters. These limitations also highlight the importance of other sensing mechanisms, such as relevant electrochemical research involving the aggregation process.

### 3.3 Plasmin Treatment of pGNP—DLS

**Figure 4:**
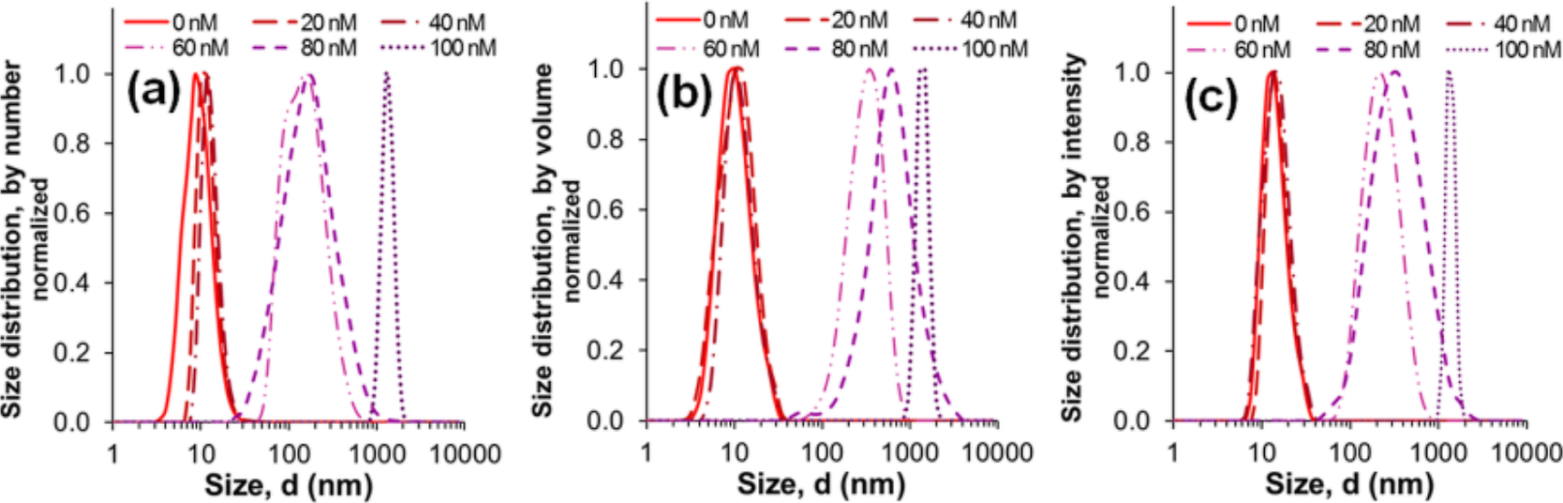
DLS analysis of enzyme-treated pGNPs. Normalized size distributions by (a) number, (b) volume, and (c) intensity of 0–100 nM plasmin-treated pGNPs, derived from dynamic light scattering measurements.

In Figure 4, DLS size measurements justify the TEM micrograph depicting ∼10 nm diameter-sized pGNPs (Figure 1b), in largely monomodal distributions presented by number (Figure 4a), volume (Figure 4b), and intensity (Figure 4c). After 0–100 nM plasmin treatment, all three representations show a definitive mean pGNP size value shift above 40 nM plasmin concentration because of aggregation. The shifted mean diameter size values, ranging from a few hundred to a thousand nm, correspond to the amount of plasmin applied and also support the absorbance data (Figure 3c,e). TEM and STEM micrographs confirm the presence of pGNP aggregates as well (Figure S6).

### 3.4 Plasmin Treatment of pGNP–Zeta Potential, Infrared Spectroscopy

**Figure 5:**
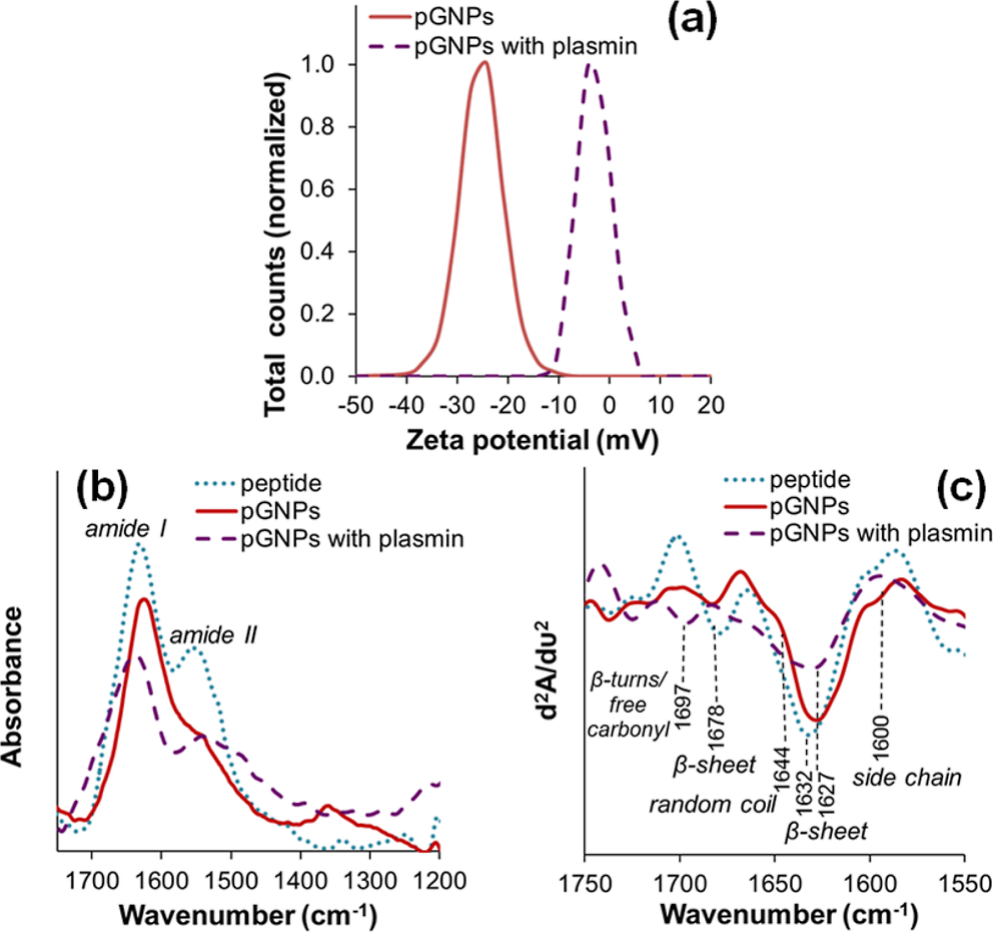
Mechanism of aggregation, presented by 100 nM plasmin-treated pGNPs. (a) Zeta potential distributions of the pGNP sample before (solid line) and after (dashed line) plasmin treatment. (b,c) Infrared comparisons of pure peptide (dotted line), peptide-stabilized pGNPs (solid line), and plasmin-treated, aggregated GNPs (dashed line): (b) amide I and amide II region of IR spectra, and (c) second derivative IR spectra of amide I stretching vibration region, characteristic for the secondary structure.

Figure 5a shows that the mean values of zeta potential distributions of pGNPs before and after 100 nM plasmin treatment were −24.1 and −4.1 mV, respectively. In the slightly alkaline solution, the peptide layer provided the particles with a significant amount of negative charge to stabilize them, which was nearly eliminated after the peptides were enzymatically fragmented. This significant loss of negative charge, i.e., the loss of electrostatic stability explains the aggregation of pGNPs.

The interpretation of FT-IR spectra of peptides and proteins usually is related to the detailed analysis of vibrations of the repeat unit of peptide bonds.^56,57^ Amide I and amide II are the two most prominent vibrational bands. The amide I, which is due almost entirely to the C=O stretching vibrations of the peptide linkage (∼80%), is the most sensitive to the secondary structural components; the frequency of amide I components correlates to different secondary elements. The results of the amide I band deconvolution by second derivative spectra are presented in Figure 5b.

The pure peptide exhibits the main band component at 1632 cm^–1^, which together with the high wavenumber counterpart at 1678 cm^–1^ is characteristic for the β-sheet structure.^58,59^ The shoulder around 1600 cm^–1^ belongs to side chain vibrations (likely ring vibration of tyrosine). Regarding the GNP, the slight shift from 1632 to 1627 cm^–1^ might be a consequence of peptide binding to the surface of pGNPs. Nevertheless, no drastic change in the secondary structure was observed.

As to the aggregated pGNPs, the feature of second derivative IR spectra is less defined, suggesting a loss of secondary structure organization, as shown in Figure 5c. Besides the main band component at 1627 cm^–1^, related to surface-bonded β-sheets, the broad derivative band has a shoulder around 1644 cm^–1^, characteristic of a disordered conformation. Moreover, the band component at 1697 cm^–1^ might belong to free carbonyl groups^60^ and suggest a destruction of the peptide H-bonding network. The aggregation phenomenon appears to be caused by the loss of the secondary structure induced by enzymatic cleavage.

### 3.5 Plasmin Treatment of pGNPs—Electrochemistry

The reading of desired reporting characteristic for aggregation (λ_max_) can be compromised by aggregation itself (Figure S5), and consequently, it is essential to use other techniques, such as electrochemistry, to determine the extent of aggregation.

**Figure 6:**
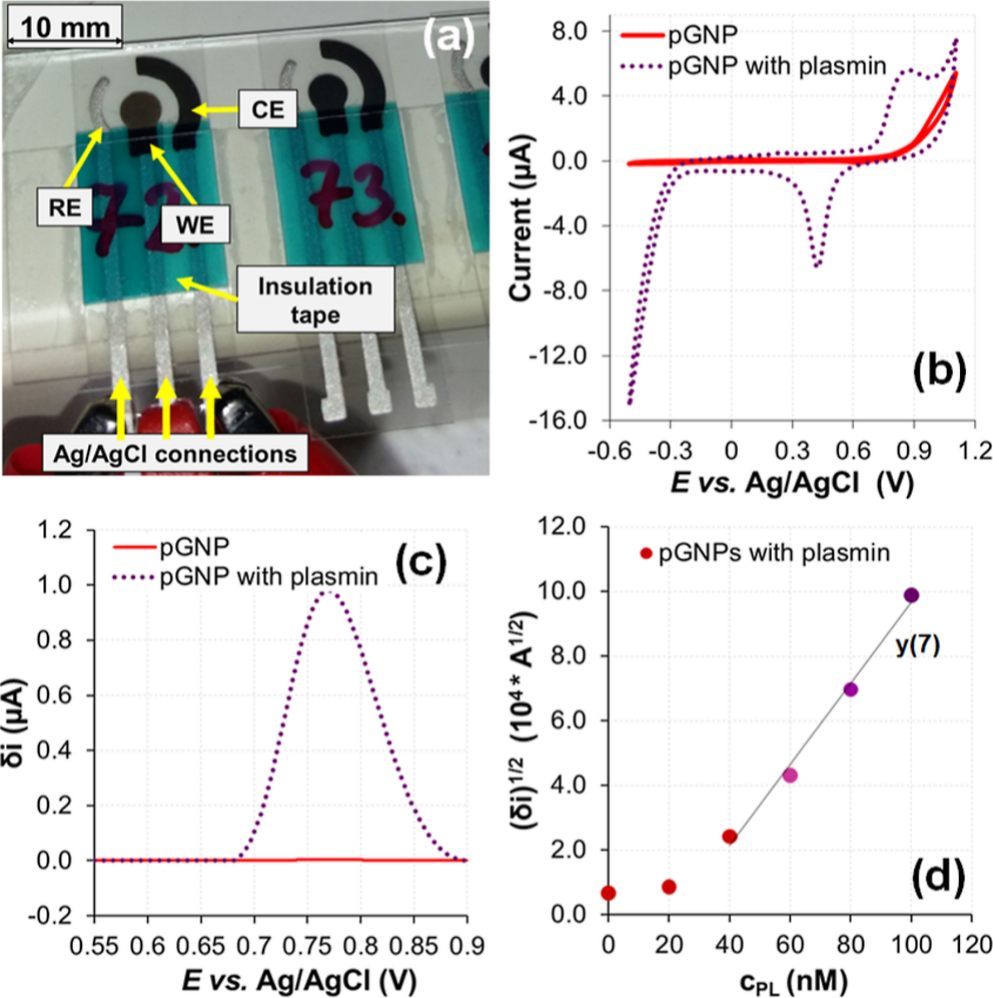
Electrochemical detection of pGNP aggregation. (a) Photograph showing the structure of graphite-based and Ag/AgCl-based screen-printed electrodes. (b) Cyclic and (b) anodic stripping square wave voltammograms of the untreated (solid line) and 100 nM plasmin-treated (dotted line) pGNPs system. (c) ASWV records of untreated (solid line) and plasmin-treated (dotted line) pGNP samples. (d) ASWV maximum values (δi) recorded after 0–100 nM plasmin treatment of pGNP sample triplets. (The error bars could not be presented because of the low error values.) Measurements were carried out with the original pGNP preparation solution, and potential values are defined versus Ag/AgCl reference electrode.

To develop a convenient analytical system, we fabricated the electrode structure shown in Figure 6a to evaluate the difference in the degree of gold electrodeposition from the samples with pGNPs alone and pGNPs treated with plasmin.

In the electrochemical detection of the gold species, gold was first electrodeposited onto the working electrode by applying a cathodic voltage. The resulting gold coated on the working electrode was then oxidized by scanning an anodic stripping square wave. Figure 6 illustrates the electrochemical behavior of the pGNP system. Comparison of intact and plasmin-treated pGNPs by cyclic voltammetry (Figure 6b) and by anodic stripping square wave voltammetry (Figure 6c) reveals that intact pGNPs do not display redox characteristics because of the stabilizing layer built-up by the peptides. The plasmin-treated samples produce specific and clear peaks of Au oxidation and reduction at around +0.8 and +0.4 V, respectively, when investigated with CV (as shown in Figure 6b). At the lower region of the cathodic sweep, hydrogen evolution occurs. Anodic SWVs recorded after cathodic deposition (Figure 6c) show similar behavior: only that oxidation peak of the sample shifts to the value of +0.77 V, which is comparable to the oxidation position occurring in the CV.

In Figure 6d, the anodic stripping square wave voltammograms show an ascending trend of (δ_*i*_)^1/2^ current peak heights with the concentration of the plasmin (*c*_PL_) applied to the pGNP samples. As shown, the aged pGNPs gave only very low oxidation response to CVs and SWVs, but due to the enzymatic loss or thinning of the stabilizing peptide layer, their surfaces become more prone to electronic contact with the electrode surface. As a result of destabilization, the pGNP aggregates tend to settle onto the electrode by gravity, thus giving a larger electrochemical response. Although the connection between δ_*i*_ and *c*_PL_ is not linear, Figure 6d reveals that when square roots of δ*i* values are considered, there is a linear oxidative response in the 40–100 nM region [see fitting data of *y*(7) in Table S5].

This nonlinear behavior is probably a consequence of the increasingly enlarged surface area, caused by deposits of pGNP aggregates.

The results of the electrochemical approach correspond with the 40–100 nM linear detection range of 2× diluted pGNP samples, as interpreted in Figure 3e.

## 4 Conclusions

A homogenous, one-batch colorimetric method was developed for the determination of the proteolytic enzyme activity, plasmin using a synthetic peptide with the combined cluster-forming site and with enzyme-sensitive amino acid sequence properties. The production of the assay substrate by simple mixing of gold salt and unlabeled peptide provides a method that does not require complex reaction conditions and purification steps and yet can be used for enzyme quantification in a given target concentration range. As both the preparation of peptide-stabilized GNPs and their enzyme-induced aggregation can be carried out in the same batch, the method has good potential in practical assay applications.

The one-batch method, besides its favorable simplicity from the practical point of view, draws attention to the importance of sample aging. Duration of peptide-stabilized nanoparticle formation should be taken into account, as early-stage samples can hinder the observable efficacy of the enzyme. Endpoint monitoring of peptide-stabilized nanoparticle development is important and can be carried out by spectrophotometric follow-up of the increase of the plasmonic absorbance peak, or, as an alternative in the present case, by the spectrofluorimetric monitoring of the evolution of the fluorescence signal produced by the oxidized form of tyrosine that serves as a reducing agent of gold (III)-ion.

The extent of nanoparticle aggregation as the enzyme activity signal was evaluated spectrophotometrically by the quantification of red shift of the samples’ plasmonic peak and also electrochemically by the increased redox responsiveness of the aggregates of enzymatic lysis-affected nanoparticles. The use of the electrochemical technique presented here is a novel approach to investigate proteolysis-induced nanoparticle aggregation in support of colorimetric assays. Spectrophotometric and electrochemical results were consistent and thus can be applied as comparable techniques.

The assay sensitivity could be enhanced by fine-tuning the ratio and concentrations of the peptide-stabilized nanoparticles and the proteolytic enzyme.

## Supporting Information Available

Practical summary of the homogenous (liquid) phase, GNP-based colorimetric protease activity assays; fitting data for spectrophotometric and spectrofluorimetric monitoring of the aging of pGNP samples, and for spectrophotometric and electrochemical detection of the aggregation of pGNPs; DLS size distributions of pGNPs; execution program of the electrochemical analysis; results of capillary electrophoresis; extended absorbance spectra of the plasmin-treated pGNPs; responsiveness of pGNPs upon treatment with different biomolecules; light scattering in the absorbance spectra; and transmission electron micrographs of pGNP aggregates (PDF)
