# Supplementary material for: Dual-Role Peptide with Capping and Cleavage Site Motifs in Nanoparticle-Based One-Pot Colorimetric and Electrochemical Protease Assay
Source: ACS Omega. 2023 Jun 9;8(25):22556–66. doi: 10.1021/acsomega.3c00771 (PMC10308550; doi:10.1021/acsomega.3c00771)
Supplement: Supplementary file 1 — ao3c00771_si_001.pdf [file ao3c00771_si_001.pdf]

Dual-role peptide with capping and cleavage site motifs in nanoparticle-based one-pot colorimetric and electrochemical protease assay

Supplementary material

Tamás Szabó†,, István Bakos†, Barbara Vrbovszki†, Itthipon Jeerapan‡, ɔ, Péter Pekker♯,*

Judith Mihály†, Krisztina Németh†, Joseph Wang‡, Zsófia Keresztes†

† Research Centre for Natural Sciences, Magyar tudósok körútja 2., 1117 Budapest, Hungary

‡ Laboratory of Nano-Bioelectronics, Department of Nanoengineering, Jacobs School of Engineering, University of California San Diego, La Jolla, CA, 92093, USA

ɔ Division of Physical Science and Center of Excellence for Trace Analysis and Biosensor, Prince of Songkla University, Hat Yai 90110, Thailand

♯ Nanolab, Research Institute of Biomolecular and Chemical Engineering, University of Pannonia, Egyetem u. 10., 8200 Veszprém, Hungary

*Corresponding author

Email address: szabo.84.tamas@ttk.hu (T. Szabó)

Table S1. A practical summary of the homogenous (liquid) phase, GNP-based colorimetric protease activity assays

'A' **Protease enzymes**	'B' **Enzyme substrates**	'C' **Source of GNPs**	'D' **State of GNPs after assaying**	'E' **Assay-ing steps #**	'F' **Destabilization of GNPs**[16]	'G' **Notes regarding labor-intensity**	'H' **Linear range (LR)**	'I' **Limit of detect. (LOD)**	**Ref.#**
**anthrax lethal factor**	c-C(S-Ac)LRRRRVYPYP-nor-LELC(S-Ac)-OH	citrate[7]	inhibited aggregation[10]	2[14]	crosslinking, chemical	GNP preparation; two assaying steps	<230 nM	25 nM	(1)
**β-secretase**	Ac-EVNLDAHFWADR	Au solution[8]	generation[11]	3	competitive reduction of Au and Cu by ascorbic acid	multiple steps	N/A	0.1 nM	(2)
**botulinum toxin A**	SNAP-25[4]	ready-made[9]	aggregation[12]	2	cleaved layer, + NaCl, charge loss	two assaying steps + developer salt	250-1500 /600-2000 fg/mL	373/278 fg/mL	(3)
**botulinum toxin A**	Cys-PEG$_{11}$-SNKTRIDEANQRAT KNorL-biotin (mimicked SNAP-25)	citrate	inhibited aggregation	2	crosslinking with neutravidine-GNPs	preparation, functionalization and purification of GNPs; two batches of GNPs, two assaying steps	N/A	5-0.1 nM	(4)
**botulinum toxin A**	SNAptide: biotin-KTRIDEANQRATKN-Cys (mimicked SNAP-25:)	ready-made	aggregation	2	crosslinking, complexation with Me^{n+}	modification and purification of GNPs; complex assaying	10 ng/mL - 1 µg/mL	0.25 ng/mL (1.67 pM)	(5)
**caspase-3**	DDDEVDGRRRR-Mercaptopropionic acid	citrate	aggregation	1	cleaved layer, fragment adsorption, charge loss	preparation, modification and purification of GNPs	27.68-129.15 pM (fluoresc.)	18 pM (fluoresc.)	(6)
**caspase-3**	GDEVDCCR	citrate	aggregation	2	fragment adsorption, charge loss	GNP preparation; two assaying steps	N/A	0.01-0.005 µg/mL	(7)
**cathepsin B**	N,N′-diBoc-dityrosine-glycine-phenylalanine-3-(methylthio)-propylamine	citrate	aggregation	2	fragment adsorption, charge loss	GNP preparation; two assaying steps	N/A	5/10 nM	(8)
**DPP-IV**[1]	RPR	citrate	inhibited aggregation	2	crosslinking, secondary bonds, charge loss	GNP preparation; two assaying steps	0.1-50/ 0.001-0.5 mU/mL (col./el.)	70/0.55 µU/mL (col./el.)	(9)

**DPP-IV**	GPDC,<br>VP-Ethylene diamine-DC	citrate	aggregation	1[15]	cleaved layer,<br>loss of steric barrier	preparation,<br>modification and purification of GNPs	0-30/<br>0-12 mU/mL	1.2/1.5 mU/mL	(10)
**IgA1P[2]**	(humane) IgA1[5]	citrate	aggregation	2	fragment adsorption, charge loss	GNP preparation;<br>two assaying steps	<0.05 mg/mL	N/A	(11)
**MMP-1[3]**	gelatin	citrate	aggregation	1	cleaved layer,<br>MCH[17]-attraction	preparation,<br>modification and purification of GNPs	N/A	N/A	(12)
**MMP-1**	collagen, casein	citrate	aggregation	1	cleaved layer,<br>MCH-attraction	preparation,<br>modification and purification of GNPs	100-700 ng/mL	N/A	(13)
**MMP-2[3]**	EEEEGPLGLAGGC	citrate	aggregation	2	ligand exchange,<br>charge loss	GNP preparation;<br>two assaying steps	<70 nM	5 nM	(14)
**MMP-2**	gelatin	citrate	aggregation	1	cleaved layer,<br>MCH-attraction	preparation,<br>modification and purification of GNPs	20-600 ng/mL	20 ng/mL	(12)
**MMP-2**	collagen, casein	citrate	aggregation	1	cleaved layer,<br>MCH-attraction	preparation,<br>modification and purification of GNPs	100-700 ng/mL	N/A	(13)
**MMP-2 : MMP-9 (1:1)**	gelatin	citrate	aggregation	1	cleaved layer + MCH,<br>MCH-attraction	preparation,<br>modification and purification of GNPs,<br>+ developer MHC	1.85-148 ng/mL	1.85 ng/mL	(15)
**MMP-7[3]**	gelatin	citrate	aggregation	1	cleaved layer,<br>MCH-attraction	preparation,<br>modification and purification of GNPs	N/A	N/A	(12)
**MMP-7**	collagen, casein	citrate	aggregation	1	cleaved layer,<br>MCH-attraction	preparation,<br>modification and purification of GNPs	100-700 ng/mL	N/A	(13)
**MMP-7**	H_6-GPLGMRGL,<br>H_6-GPLGMRGL-H_6	citrate	aggregation	1,<br>mix-and-detect	ligand exchange, crosslinking in presence of Me^{n+}	preparation,<br>modification and purification of GNPs,<br>+chelators, + Me^{n+}	3-52 nM	10 nM	(16)
**MMP-7**	H_6-GPLGMRGL-H_6	citrate	inhibited aggregation	2	crosslinking, complexation with Me^{n+} and fragments	preparation and modification of GNPs<br>+ Me^{n+},	N/A	0.41/0.12 nM	(17)
**MMP-7**	NAADLEKAIEALEK<br>HLEAKGPCDAAQLE<br>KQLEQAFEAFERAG	citrate	aggregation	1	cleaved layer,<br>charge loss	preparation,<br>modification and purification of GNPs	N/A	0.082/1.25 µg/mL	(18)

**MMP-7**	NAADLEKAIEALEK HLEAKGPCDAAQLE KQLEQAFEAFERAG	citrate	aggregation	1	cleaved layer, charge loss	preparation, modification and purification of GNPs	N/A	3.1/12.5 (165/650) µg/mL (nM)	(19)
**MMP-7**	NAADLEKAIEA-LEKHLEAKGPCDAA Q-LEKQLEQAFEAFER AG	citrate	aggregation	1	cleaved layer, charge loss	preparation, modification and purification of GNPs	<2 µg/mL	5 nM (0.1 µg/mL)	(20)
**MMP-9[3]**	gelatin	citrate	aggregation	1	cleaved layer, MCH-attraction	preparation, modification and purification of GNPs	N/A	N/A	(12)
**plasmin**	fibrinogen	citrate	aggregation	1	cleaved layer, + NaCl, charge loss	preparation, modification and purification of GNPs + developer	N/A	0.4 nM	(21)
**plasmin**	CCYGGTFKGGGGG GR	in situ prepared	adsorption	1	cleaved layer, charge loss	preparation of GNPs	40-100/ 70-500 nM	30-40 nM	This work
**thermolysin**	Fmoc[6]-GFC-NH_2	N/A	dissociation[13]	1	crosslinking, π-π interactions	previous functionalization and aggregation of GNPs	N/A	90 zg/mL 10 ag/mL	(22)
**thermolysin**	SH–Ph–CH2–GGGFGGK(NH2)–CO–NH_2	ready-made	dissociation	1	crosslinking, primary bonds	modification and purification of GNPs	N/A	34.1 ng/mL	(23)
**thrombine**	Ac-C(S-Ac)G*D*FPRGC(S-Ac)-OH	citrate	inhibited aggregation	2	crosslinking, primary bonds	GNP preparation; two assaying steps	<45 nM	5 nM	(1)
**thrombine**	fibrinogen	citrate	aggregation	1	crosslinking, primary bonds	preparation, modification and purification of GNPs + centrifugation	0.1-10 pM	0.04 pM	(24)
**trypsin**	gelatin	citrate	aggregation	1	cleaved layer, MCH-attraction	preparation, modification and purification of GNPs	0.125-125 U	0.0125 U	(12)
**trypsin**	R_6	citrate	inhibited aggregation	2	crosslinking, electrostatic	GNP preparation; two assaying steps	<3 µM	1.6 ng/mL	(25)
**trypsin**	EEEEGLLGALGKC	citrate	aggregation	2	ligand exchange, charge loss	GNP preparation; two assaying steps	N/A	5 nM	(14)
**trypsin**	YHPQMNPYTKAGG GC	citrate	inhibited aggregation	2	crosslinking, secondary bonds	GNP preparation; two assaying steps	<8 nM	0.5 nM	(26)

**trypsin**	β-casein	citrate	aggregation	1	cleaved layer MCH-attraction	preparation, modification and purification of GNPs	<1 nM	0.42 nM	(27)

[1] DPP-IV: dipeptidyl peptidase-4

[2] IgA1P: protease of immunoglobulin A (isotype 1)

[3] MMP-1,2,7 and 9: matrix metalloproteinases

[4] SNAP-25: synaptosomal-associated protein, 25 kDa

[5] IgA1: an isotype of immunoglobulin A

[6] Fmoc-: *N*-(fluorenyl-9- methoxycarbonyl)-

[7] citrate: reduced from the solution of Au-salt with citrate

[8] Au solution

[9] ready-made: purchased and later modified

[10] inhibited aggregation: when the enzyme is added, aggregation of GNPs induced by intact substrates are inhibited.

[11] generation: generation of GNPs are induced by the intact/cleaved substrates of the enzyme.

[12] aggregation: GNPs aggregate upon the activity of the enzyme, due to the generated substrate fragments. The process involves the destabilization of the GNPs surface layer via ligand exchange, fragment adsorption on the surface of GNPs, or different types of crosslinking induced by the product of the enzyme reaction.

[13] dissociation: formerly aggregated or crosslinked GNPs become free after enzymatic cleavage of the bonds.

[14] 2 steps reaction, usually the first step is the reaction of the enzyme and the substrate, the second is the mixing of the cleaved substrate with the GNPs, also called ‘mix-and-detect’ method.

[15] 1 step reaction, usually the enzyme is added to the GNPs that are modified with the substrate of the enzyme.

[16] Destabilization of GNPs:

- crosslinking, when a third party is involved, forming a bridge between GNPs (or their modifying layer): primary bonds; MCH-attraction (interaction between -OH group and GNP surface); electrostatic; *π*-*π* interactions; complexation in the presence of chelator and some metallic ion Me^{n+}; other secondary bonds.
- individual destabilization, usually involves the charge loss of the electrically charged modifying layer of GNPs: partial removal of the charged surface lyer; adsorption of counter-charged fragments on the charged surface layer; ligand exchange on the charged surface layer; addition of salt; other type is the removal of surface layer that poses a non-charged steric barrier

Both strategies are based on the products (cleaved fragments of the enzyme substrate) of the proteolytic reaction, and can be combined.

[17] MCH: 6-mercaptohexan-1-ol

Table S2. Fitting data for the spectrophotometric and spectrofluorimetric monitoring of the ageing of pGNP samples (related to Fig. 2c).

**Measuerement**	**Age of pGNP sample (h)**	**Fitted trendline***	**x^2**	**x**	**C**	**R^2**
**Absorbance**	0-672	y(1)	-3E-06	+0.0043	+0.2395	0.993
	672-1776	y(2)	+6E-08	+0.0004	+1.3713	0.9937
**Fluorescence**	0-672	y(3)	-2E-07	+0.0069	+0.2889	0.9948
	672-1776	y(4)	0	+0.0007	+4.4984	0.8956

*All the fittings are of 2nd order polynomial;

Table S3. Mean values of pGNP size distributions obtained form DLS measurements (related to Fig. 2d).

**Age of pGNP sample (h)**	**Mean diameter by Number (nm)**	**Mean diameter by Volume (nm)**	**Mean diameter by Intensity (nm)**
**672**	8.7	10.1	13.5
**1776**	8.7	11.1	15.7

Table S4. Fitting data for the spectrophotometric detection of aggregation of pGNP samples (related to Fig. 3d, e).

**Linear sensing range of plasmin (nM)**	**Dilution of pGNP sample**	**Fitted trendline***	**x**	**C**	**R^2**
**70-500**	-	y(5)	0.0458	524.27	0.9878
**40-100**	2x	y(6)	0.1032	523.57	0.9885

*All the fittings are of 2nd order polynomial;

Table S5. Fitting data for the electrochemical detection of aggregation of pGNP samples (related to Fig. 6c).

Linear sensing range of plasmin	Dilution of pGNP sample	Fitted trendline*	x	C	R^2
**40-100 nM**	2x	y(7)	0.1252	2.8668	0.9914

*The fitting is of 2nd order polynomial;

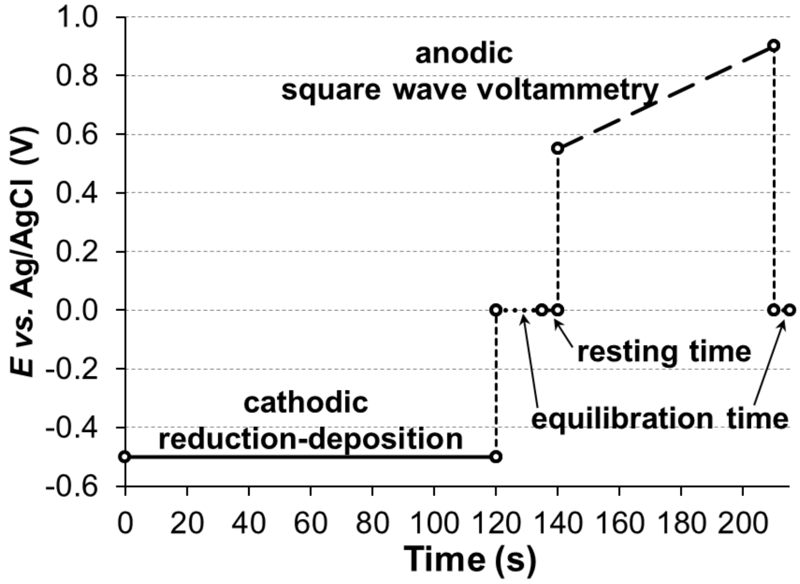

Figure S1. Execution program of cathodic accumulation followed by anodic stripping square wave voltammetry.

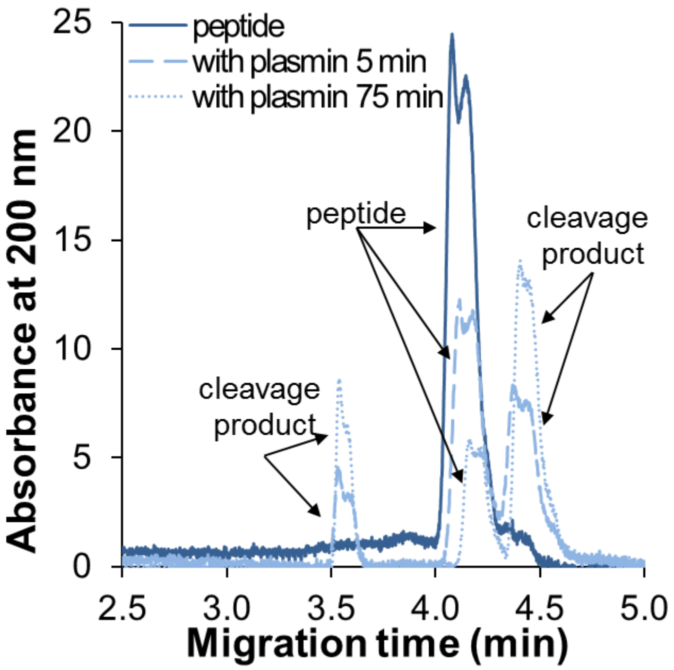

Figure S2. Capillary electrophoresis showed that the endpoint conversion of 100 µM peptide by 1 µM plasmin was around 70-75% within 75 min at 25o C. Both the two cleavage products (migration times: 3.5 min and 4.3 min) and the substrate peptide (4.0 min) separated well in the electropherograms and each could be detected 5 minutes after of addition of plasmin to the reaction mixture.

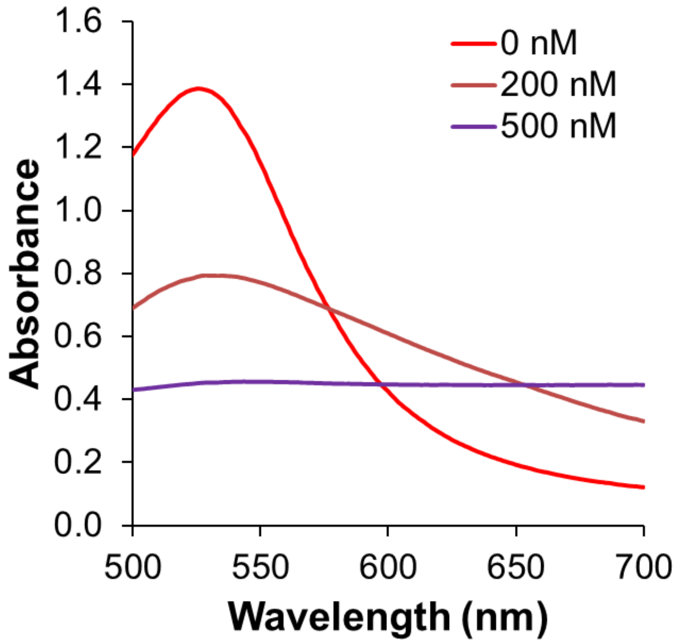

Figure S3. Extended absorbance spectra of pGNP samples after the addition of 0-500 nM plasmin. The spectra represent only one characteristic peak between 500 and 550 nm.

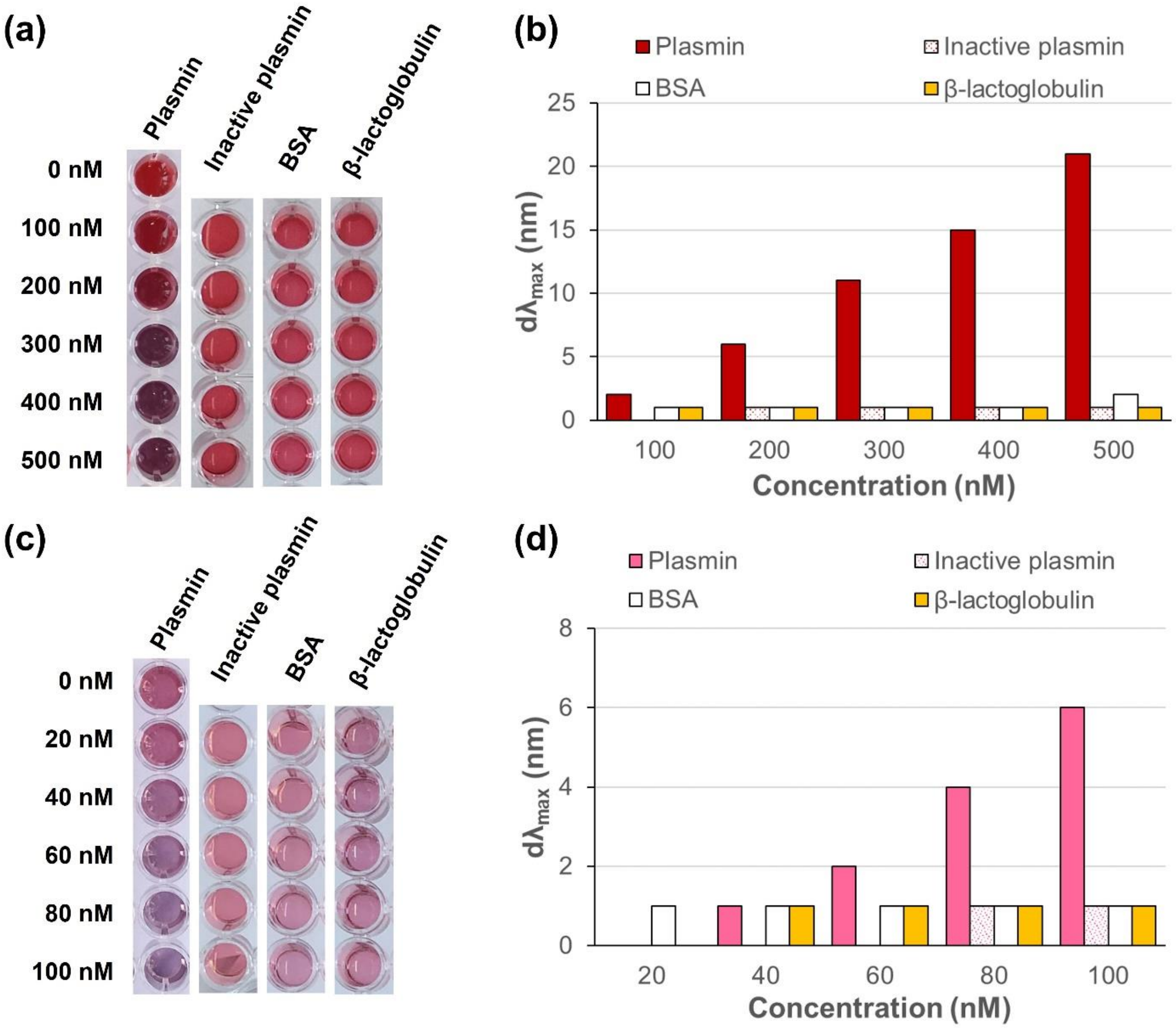

Figure S4. Representative comparison of the responsiveness of pGNP assay substrate upon treatment with different biomolecules: plasmin, thermally deactivated plasmin, bovine serum albumin and β-lactoglobulin. **(a)** and **(b):** color change and absorbance maxima change of 0-500 nM biomolecules-treated pGNPs, respectively. **(c)** and **(d):** color change and absorbance maxima change of 2x PBS-diluted, 0-100 nM biomolecules-treated pGNPs, respectively.

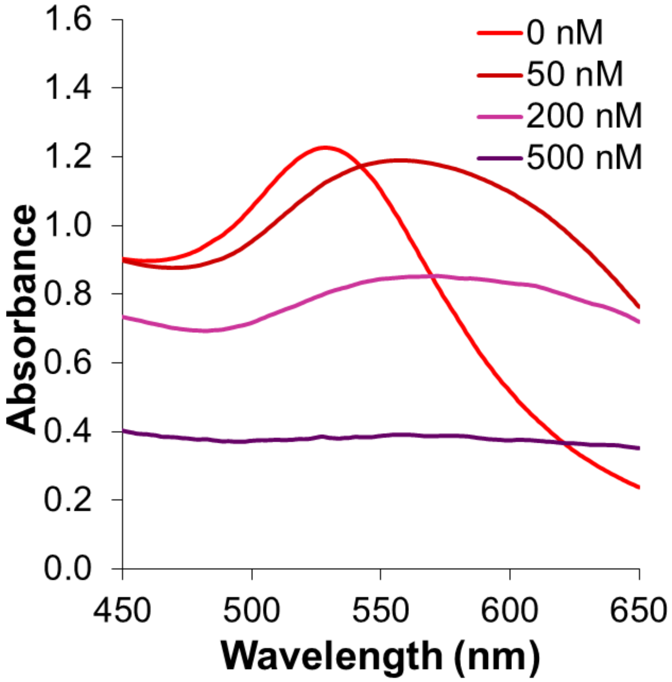

Figure S5. Absorbance spectra of pGNP samples after the addition of 0-500 nM plasmin. Light scattering appears at 500 nanomolar dose due to heavy aggregation of pGNPs caused by excessive plasmin activity.

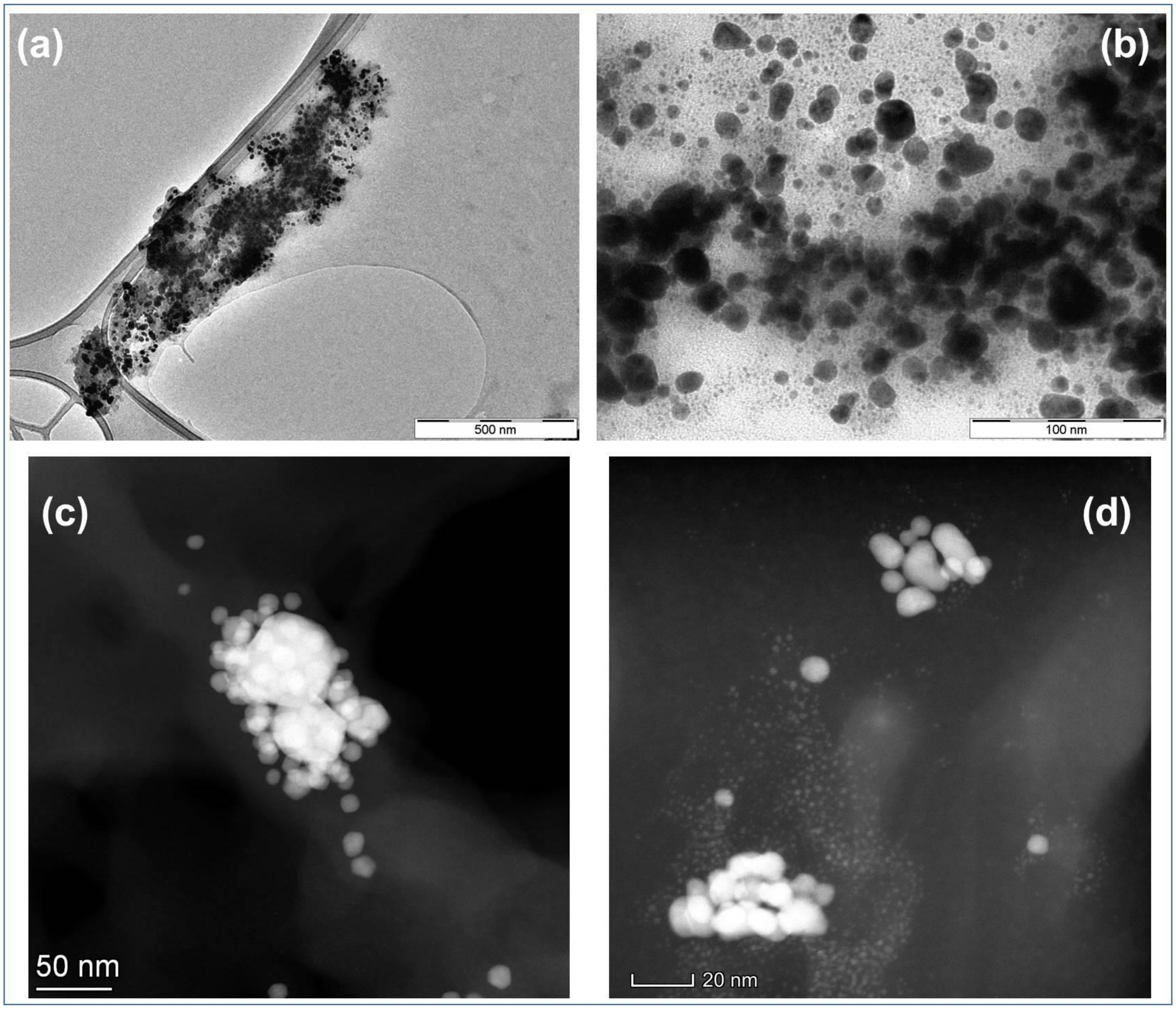

Figure S6. Transmission electronmicrographs of aggregated pGNP particles after 500 nM plasmin treatment. **(a)** and **(b):** TEM micrographs with scales representing 500 nm and 100 nm, respectively. **(c)** and **(d):** STEM with HAADF detection mode micrographs with scales representing 50 nm and 20 nm, respectively.

REFERENCES

(1) Guarise, C.; Pasquato, L.; De Filippis, V.; Scrimin, P. Gold Nanoparticles-Based Protease Assay. Proc. Natl. Acad. Sci. U. S. A. 2006. https://doi.org/10.1073/pnas.0509372103.

(2) Liu, L.; Deng, D.; Wang, Y.; Song, K.; Shang, Z.; Wang, Q.; Xia, N.; Zhang, B. A Colorimetric Strategy for Assay of Protease Activity Based on Gold Nanoparticle Growth Controlled by Ascorbic Acid and Cu(II)-Coordinated Peptide. Sensors Actuators, B Chem. 2018. https://doi.org/10.1016/j.snb.2018.03.116.

(3) Halliwell, J.; Gwenin, C. A Label Free Colorimetric Assay for the Detection of Active Botulinum Neurotoxin Type a by SNAP-25 Conjugated Colloidal Gold. Toxins (Basel). 2013, 5 (8), 1381–1391. https://doi.org/10.3390/toxins5081381.

(4) Liu, X.; Wang, Y.; Chen, P.; Wang, Y.; Zhang, J.; Aili, D.; Liedberg, B. Biofunctionalized Gold Nanoparticles for Colorimetric Sensing of Botulinum Neurotoxin A Light Chain. Anal. Chem. 2014, 86 (5), 2345–2352. https://doi.org/10.1021/ac402626g.

(5) Chen, S.; Chu, L. T.; Chen, T. H. Colorimetric Detection of Active Botulinum Neurotoxin Using Cu2+ Mediated Gold Nanoparticles Agglomeration. Sensors Actuators, B Chem. 2016, 235, 563–567. https://doi.org/10.1016/j.snb.2016.05.118.

(6) Li, J.; Li, X.; Shi, X.; He, X.; Wei, W.; Ma, N.; Chen, H. Highly Sensitive Detection of Caspase-3 Activities via a Nonconjugated Gold Nanoparticle-Quantum Dot Pair Mediated by an Inner-Filter Effect. ACS Appl. Mater. Interfaces 2013, 5 (19), 9798–9802. https://doi.org/10.1021/am4029735.

(7) Pan, Y.; Guo, M.; Nie, Z.; Huang, Y.; Peng, Y.; Liu, A.; Qing, M.; Yao, S. Colorimetric Detection of Apoptosis Based on Caspase-3 Activity Assay Using Unmodified Gold Nanoparticles. Chem. Commun. 2012, 48 (7), 997–999. https://doi.org/10.1039/c1cc15407a.

(8) Kim, C. J.; Lee, D. I.; Kim, C.; Lee, K.; Lee, C. H.; Ahn, I. S. Gold Nanoparticles-Based Colorimetric Assay for Cathepsin B Activity and the Efficiency of Its Inhibitors. Anal. Chem. 2014, 86 (8), 3825–3833. https://doi.org/10.1021/ac4039064.

(9) Xia, N.; Wang, X.; Wang, X.; Zhou, B. Gold Nanoparticle-Based Colorimetric and Electrochemical Methods for Dipeptidyl Peptidase-IV Activity Assay and Inhibitor Screening. Materials (Basel). 2016, 9 (10). https://doi.org/10.3390/ma9100857.

(10) Aldewachi, H. S.; Woodroofe, N.; Turega, S.; Gardiner, P. H. E. Optimization of Gold Nanoparticle-Based Real-Time Colorimetric Assay of Dipeptidyl Peptidase IV Activity. Talanta 2017, 169 (March), 13–19. https://doi.org/10.1016/j.talanta.2017.03.039.

(11) Garner, A. L.; Fullagar, J. L.; Day, J. A.; Cohen, S. M.; Janda, K. D. Development of a High-Throughput Screen and Its Use in the Discovery of Streptococcus Pneumoniae Immunoglobulin A1 Protease Inhibitors. J. Am. Chem. Soc. 2013, 135 (27), 10014–10017. https://doi.org/10.1021/ja404180x.

(12) Chuang, Y. C.; Li, J. C.; Chen, S. H.; Liu, T. Y.; Kuo, C. H.; Huang, W. T.; Lin, C. S. An Optical Biosensing Platform for Proteinase Activity Using Gold Nanoparticles. Biomaterials 2010, 31 (23), 6087–6095. https://doi.org/10.1016/j.biomaterials.2010.04.026.

(13) Chuang, Y. C.; Huang, W. T.; Chiang, P. H.; Tang, M. C.; Lin, C. S. Aqueous Zymography Screening of Matrix Metalloproteinase Activity and Inhibition Based on Colorimetric Gold Nanoparticles. Biosens. Bioelectron. 2012, 32 (1), 24–31. https://doi.org/10.1016/j.bios.2011.11.002.

(14) Chen, G.; Xie, Y.; Zhang, H.; Wang, P.; Cheung, H. Y.; Yang, M.; Sun, H. A General Colorimetric Method for Detecting Protease Activity Based on Peptide-Induced Gold Nanoparticle Aggregation. RSC Adv. 2014, 4 (13), 6560–6563. https://doi.org/10.1039/c3ra46493h.

(15) Nossier, A. I.; Mohammed, O. S.; Fakhr El-deen, R. R.; Zaghloul, A. S.; Eissa, S. Gelatin-Modified Gold Nanoparticles for Direct Detection of Urinary Total Gelatinase Activity: Diagnostic Value in Bladder Cancer. Talanta 2016, 161, 511–519. https://doi.org/10.1016/j.talanta.2016.09.015.

(16) Kim, G. B.; Kim, K. H.; Park, Y. H.; Ko, S.; Kim, Y. P. Colorimetric Assay of Matrix Metalloproteinase Activity Based on Metal-Induced Self-Assembly of Carboxy Gold Nanoparticles. Biosens. Bioelectron. 2013, 41 (1), 833–839. https://doi.org/10.1016/j.bios.2012.10.025.

(17) Kim, G. B.; Lee, J. O.; Kim, Y. P. Graying the Self-Assembly of Gold Nanoparticles for Improved Enzyme Activity Assays. Sensors Actuators, B Chem. 2017, 246, 271–277. https://doi.org/10.1016/j.snb.2017.02.067.

(18) Chen, P.; Liu, X.; Goyal, G.; Tran, N. T.; Shing Ho, J. C.; Wang, Y.; Aili, D.; Liedberg, B. Nanoplasmonic Sensing from the Human Vision Perspective. Anal. Chem. 2018, 90 (7), 4916–4924. https://doi.org/10.1021/acs.analchem.8b00597.

(19) Goyal, G.; Palaniappan, A.; Liedberg, B. Protease Functional Assay on Membrane. Sensors Actuators, B Chem. 2020, 305 (November 2019), 127442. https://doi.org/10.1016/j.snb.2019.127442.

(20) Chen, P.; Selegård, R.; Aili, D.; Liedberg, B. Peptide Functionalized Gold Nanoparticles for Colorimetric Detection of Matrilysin (MMP-7) Activity. Nanoscale 2013, 5 (19), 8973–8976. https://doi.org/10.1039/c3nr03006g.

(21) Jian, J. W.; Chiu, W. C.; Chang, H. T.; Hsu, P. H.; Huang, C. C. Fibrinolysis and Thrombosis of Fibrinogen-Modified Gold Nanoparticles for Detection of Fibrinolytic-Related Proteins. Anal. Chim. Acta 2013, 774, 67–72. https://doi.org/10.1016/j.aca.2013.02.024.

(22) Laromaine, A.; Koh, L.; Murugesan, M.; Ulijn, R. V.; Stevens, M. M. Protease-Triggered Dispersion of Nanoparticle Assemblies. J. Am. Chem. Soc. 2007, 129 (14), 4156–4157. https://doi.org/10.1021/ja0706504.

(23) Liu, R.; Aw, J.; Teo, W.; Padmanabhan, P.; Xing, B. Novel Trimethyl Lock Based Enzyme Switch for the Self-Assembly and Disassembly of Gold Nanoparticles. New J. Chem. 2010, 34 (4), 594–598. https://doi.org/10.1039/b9nj00776h.

(24) Chen, C. K.; Huang, C. C.; Chang, H. T. Label-Free Colorimetric Detection of Picomolar Thrombin in Blood Plasma Using a Gold Nanoparticle-Based Assay. Biosens. Bioelectron. 2010, 25 (8), 1922–1927. https://doi.org/10.1016/j.bios.2010.01.005.

(25) Xue, W.; Zhang, G.; Zhang, D. A Sensitive Colorimetric Label-Free Assay for Trypsin and Inhibitor Screening with Gold Nanoparticles. Analyst 2011, 136 (15), 3136–3141. https://doi.org/10.1039/c1an15224f.

(26) Ding, X.; Ge, D.; Yang, K. L. Colorimetric Protease Assay by Using Gold Nanoparticles and Oligopeptides. Sensors Actuators, B Chem. 2014, 201, 234–239. https://doi.org/10.1016/j.snb.2014.05.014.

(27) Piovarci, I.; Melikishvili, S.; Tatarko, M.; Hianik, T.; Thompson, M. Detection of Sub-Nanomolar Concentration of Trypsin by Thickness-Shear Mode Acoustic Biosensor and Spectrophotometry. Biosensors 2021, 11 (4). https://doi.org/10.3390/bios11040117.
